# Awareness and knowledge of the common features of inflammatory back pain among primary care physicians in the western region of Saudi Arabia

**DOI:** 10.1097/MD.0000000000031626

**Published:** 2022-10-28

**Authors:** Roaa Aljohani, Noha Barradah, Amnah Kashkari

**Affiliations:** a Department of Medicine, College of Medicine, Taibah University, Madinah, Saudi Arabia; b Department of Medicine, Taibah University, Medina, Saudi Arabia.

**Keywords:** ankylosing spondylitis, inflammatory back pain, knowledge, mechanical back pain, primary care physicians

## Abstract

Often, there is a delay in the diagnosis of inflammatory back pain (IBP) in the primary care setting. This may be attributed to the inability of healthcare providers to distinguish between inflammatory and mechanical back pain. This study aimed to evaluate primary care physicians’ current practices for assessing patients with IBP using clinical, radiographic, and laboratory tests. A questionnaire-based survey was emailed to all primary care physicians in the western region of Saudi Arabia by the Saudi Commission of Health Specialists from February to May 2021. The questionnaire included data about axial spondyloarthropathy based on the Calin, Berlin, and European Spondyloarthropathy Study Group criteria. A total of 103 primary care physicians responded who represented around 24% of primary care physicians at primary healthcare. The most often perceived IBP symptoms include a response to NSAIDs, morning stiffness lasting >30 minutes, age of onset <45 years old, duration of back pain >3 months, and improvement with exercise. The most frequently questioned patient or family history conditions were peripheral arthritis (92.2%), family history of spondyloarthritis (83.5%), and inflammatory bowel disease (97.6%). The most-reported investigations were CRP/ESR (86.4%) and spinal radiography (66%). For treatment of IBP, NSAIDs were most prescribed (48.6%), followed by physiotherapy (45.6%) and disease-modifying anti-rheumatic drugs (41.7%). Primary care physicians were more confident in management of mechanical back pain than IBP (*P* < .001). Primary care physicians have good knowledge of IBP symptoms but not of disease-specific features and modest confidence in evaluating patients with IBP, indicating the need for educational programs and a more effective, feasible referral strategy.

## 1. Introduction

Saudi Arabia’s healthcare system is overseen by the Ministry of Health, which offers free healthcare to both Saudi and non-Saudi public sector employees. There are three levels of free healthcare services in Saudi Arabia (primary, secondary, and tertiary), with primary healthcare (PHC) serving as the entry point into the healthcare system, providing curative and preventative care. Physicians providing PHC can refer patients to a specialist at a higher-level facility if they require a service that is not provided at the primary-care level. With the increasing population, there is a growing improvement in PHC services, but this still does not meet patients’ needs and expectations.

There is still a scarcity of physicians, and the majority of health care providers in Saudi Arabia are expatriates, which contributes to a significant turnover rate and an unstable workforce. Moreover, PHC is provided by family physicians or general practitioners with differing levels of training and experiences. A family physician is a qualified doctor who specialized in family medicine after graduating from medical school, while a general practitioner is a doctor with qualifications from a medical school who chose to work in primary care.

Back pain is one of the most frequent symptoms that leads patients to seek PHC. Recent global guidelines have emphasized that patients with low back pain (LBP) should receive therapy at the PHC level, as only a minority of cases require referral to a specialist. However, there are no existing clinical recommendations or established pathways of care for patients with LBP in Saudi Arabia. As a result, it is critical to understand when a patient should be referred to secondary or tertiary care centers and what investigations or treatments patients may require that are not available in a PHC setting.

Chronic back pain, one of the most common symptoms encountered in outpatient clinics, affects a substantial proportion of the population and results in considerable patient impairment and usage of healthcare resources.^[1]^ Approximately 80% of the population develop an episode of back pain in their lifetime. Although the condition of most people with back pain improves over time, a significant percentage of the population continues to experience chronic symptoms.^[2]^ Axial spondyloarthritis (axSpA), a chronic inflammatory rheumatic illness affecting mostly the spine and sacroiliac joints, is a significant, though underdiagnosed, cause of persistent low back pain.^[3,4]^ SpA is a broad term that refers to various chronic inflammatory rheumatic disorders, such as ankylosing spondylitis (AS), psoriatic arthritis, enteropathic spondylitis, and non-radiographic axial SpA.^[5]^ Approximately 5% of primary care patients with chronic back pain have AS or other axial spondyloarthritides.^[2]^ The condition often begins in early adulthood (between the ages of 20 and 30 years), and over 90% of patients are less than 45 years old at the disease onset.^[6]^

The diagnosis of axSpA can be challenging; thus, it is critical to maintain a high index of suspicion to minimize the time of diagnosis, which presently ranges between 5 and 10 years after the onset of symptoms.^[7,8]^ Early recognition of AS can be challenging for several reasons, including mild-to-moderate symptoms upon presentation, delayed disease progression, a lack of reliable diagnostic testing, and a low prevalence.^[9]^ Although there are many reasons for the delay in diagnosing AS, recent studies have shown that a lack of general practitioner (GP) awareness contributes considerably to this delay.^[10]^ The adverse consequences of delayed diagnosis and untreated disease may diminish a patient’s quality of life and result in a lengthy medical leave and increased economic burden. In addition, delayed diagnosis may promote severe disability associated with untreated disease in its early years of development.^[11,12]^ The Assessment of SpondyloArthritis International Society (ASAS) criteria represent a substantial move forward regarding identifying individuals with axial SpA earlier in their disease course.^[13–15]^ The imaging arm requires evidence of sacroiliitis on radiography or magnetic resonance imaging, whereas the clinical component requires positive human leukocyte antigen (HLA)-B27. To fulfill the ASAS criteria, a minimum of one parameter in the imaging arm or two parameters in the clinical arm should be present, in addition to the following additional Spondyloarthritis features: inflammatory back pain (IBP), arthritis, enthesitis/uveitis, dactylitis, psoriasis, inflammatory bowel disease (IBD), good response to non-steroidal anti-inflammatory drugs (NSAIDs), positive HLA-B27, and increased C-reactive protein (CRP) level. Although the ASAS has highlighted early diagnosis as one of its goals to enhance patient care, research has shown that primary care physicians (PCPs) are unaware of the disease spectrum and treatment options.^[10,16]^ This study aimed to assess PCPs’ knowledge and confidence about IBP and its clinical approach by employing clinical symptoms, laboratory, and radiological examinations to diagnose and refer patients with IBP in primary care clinics in Saudi Arabia’s western region.

## 2. Methods

### 2.1. Study design and participants

A cross-sectional questionnaire-based survey distributed by the Saudi Commission for Health Specialties to all PCPs in Saudi Arabia’s western region was conducted from February to May 2021. All PCPs who volunteered to participate provided their informed consent and the local ethics committee was approved this study. The questionnaire was designed to collect data on the awareness of the characteristics of chronic inflammatory low back pain. The first section of the questionnaire collected demographic data, and the second section assessed the clinical knowledge and confidence levels of the respondents. The questionnaire was designed to cover various aspects of disease, including symptoms, disease features, and essential investigations based on the Calin, Berlin, and European Spondyloarthropathy Study Group criteria for axial spondyloarthropathies, which all have a similar performance in diagnosing IBP and can be used in daily clinical practice.^[17,18]^ The respondents were asked to score the significance of individual symptoms as an indicator of IBP and the same questions as indicative of mechanical back pain (MBP) (10-point scale). The PCPs were asked about IBP-associated features, such as enthesitis, peripheral arthritis, a family history of spondyloarthritis, uveitis, IBD, dactylitis, psoriasis, and genitourinary or gut infection in the last month. They were also asked questions regarding the therapies that they considered necessary (four-point scale) for patients with suspected IBP and the investigations that they thought were critical. Key important questions were the length of time for the referral process and which specialist service they would refer to in cases of suspected IBP. Additionally, respondents were questioned about their degree of confidence in managing patients with IBP versus those with MBP and what learning tools they thought would help in improving their expertise.

### 2.2. Statistical analysis

Categorical variables are described as frequencies and percentages, whereas continuous numerical variables are presented as an arithmetic mean and standard deviation. The student’s *t* test was used to compare the means of two continuous numerical variables, and a *P* value of <.05 was considered statistically significant. The data were described and analyzed using the Statistical Package for Social Sciences, version 26.

## 3. Results

**Figure 1.**
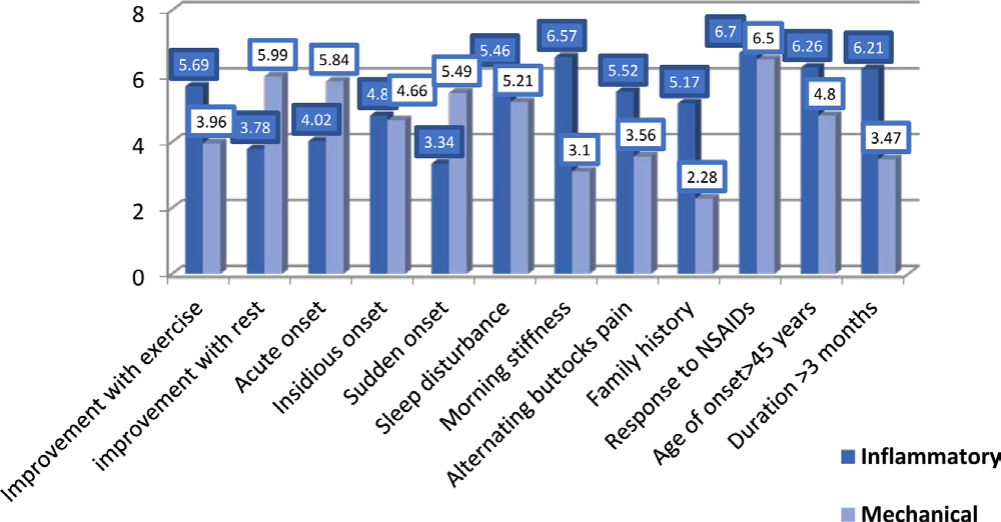
Physician’s perception of the importance of the symptoms as an indication of inflammatory or mechanical back pain (mean score out of a score ranging between 1-least important and 10-most important). NSAIDs = non-steroidal anti-inflammatory drugs.

**Figure 2.**
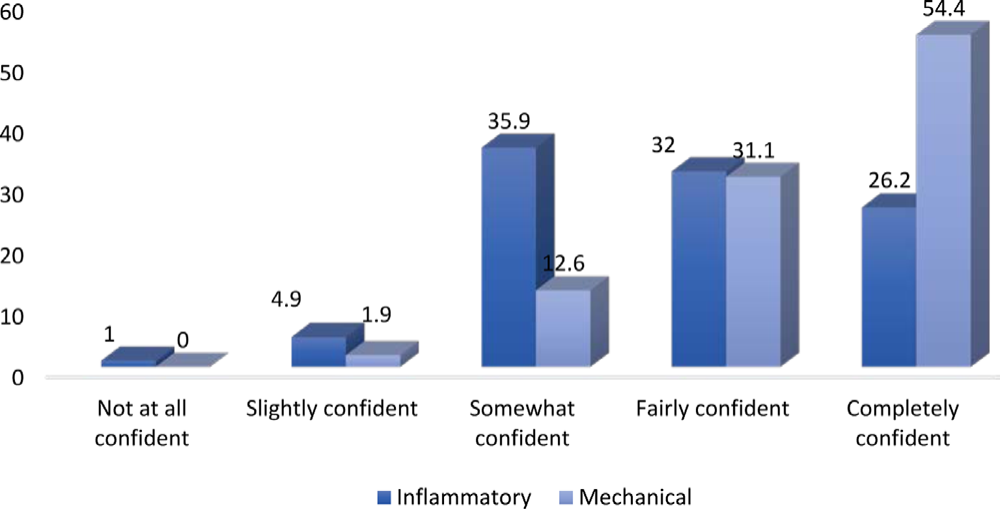
Physicians’ confidence level in assessing patients with inflammatory/mechanical back pain.

**Figure 3.**
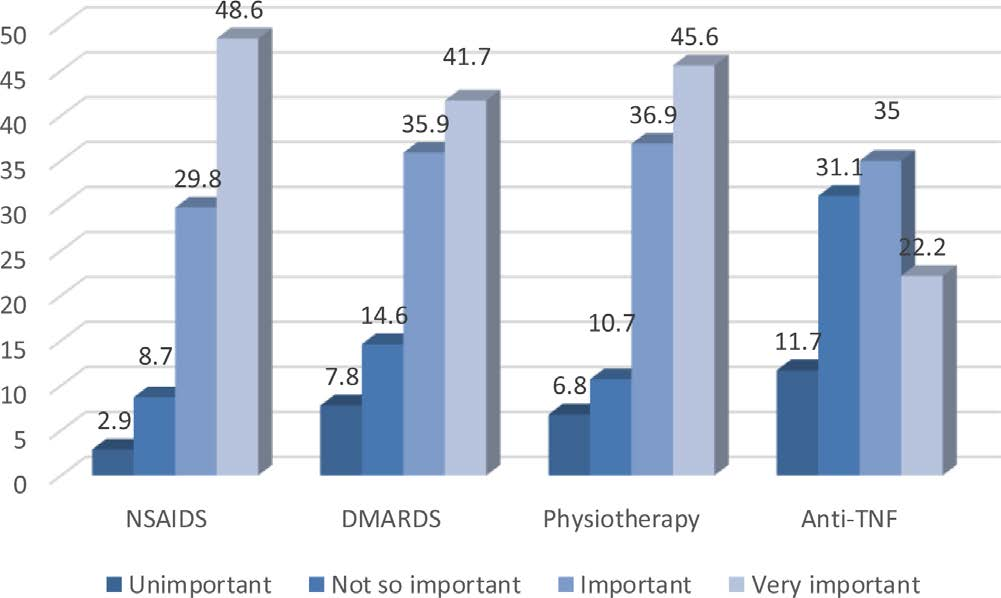
Physicians’ beliefs concerning the most important line of treatment in cases of patient with inflammatory back pain. DMARDS = disease-modifying anti-rheumatic drugs, NSAIDS = non-steroidal anti-inflammatory drugs, TNF = tumor necrosis factor.

Among the 103 PCPs who completed the questionnaires, 49 were GPs, and 54 were family physicians. The frequency of cases of back pain seen per week was as follows: 16 physicians, ≥10 cases/week, 46 physicians, 5 to 9 cases/week, and 41 physicians, <5 cases/week. Of these, 52.4% of the physicians had been practicing for > 10 years. Their demographic characteristics are summarized in Table 1. Regarding IBP symptoms, the most perceived important symptoms (mean score rated out of 10 ± SD) were the duration of back pain of >3 months (96.21 ± 3.49), response to NSAIDS (6.70 ± 3.37), morning stiffness lasting >30 minutes (6.57 ± 3.7), age of onset <45 years old (6.26 ± 3.52), and improvement with movement/exercise (5.69 ± 3.96). Regarding MBP, the most perceived important symptoms were the response to NSAIDS (6.50 ± 3.36), improvement with rest (5.99 ± 3.74), acute onset (5.84 ± 3.44), sudden onset (5.49 ± 3.66), and sleep disturbance by back pain (5.21 ± 3.81). Comparatively, the following symptoms were more statistically significant for IBP than for MBP: improvement with rest, acute onset, sudden onset, morning stiffness, alternating buttock pain, family history (*P* < .001), improvement with exercise (*P* = .002), and duration of >3 months (*P* = .005), as shown in Figure 1. The following were the most frequently asked medical/family history conditions for patients suspected of being diagnosed with IBP: peripheral arthritis (92.2%), family history of spondyloarthritis (83.5%), history of IBD (79.6%), genitourinary/gut infection in the last month (64.1%), uveitis (62.1%), psoriasis (54.4%), dactylitis (51.5%), and enthesitis (42.7%) (Table 2). The most frequently reported investigations believed to be important for patients suspected of having IBP were CRP/erythrocyte sedimentation rate (ESR) blood tests (86.4%), radiography of the spine (66%), HLA-B27 blood tests (47.6%), and radiography of the pelvis (47.6%), as presented in Table 3. Furthermore, more than half of the PCPs (54.4%) were completely confident when managing MBP cases compared with only 26.2% when managing IBP cases (*P* < .001) (Fig. 2). The average duration to refer a suspicious case of IBP to specialist services was 1 month (45.6%), 1 to 3 months (31%), and >3 months (23.3%). For IBP referrals, referrals to rheumatologists ranked first (68%), followed by orthopedic surgeons (26.2%) and physiotherapists (5.8%). The most-reported important line of treatment for patients with IBP was NSAIDS (48.6%), followed by physiotherapy (45.6%), disease-modifying anti-rheumatic drugs (41.7%), and anti-tumor necrosis factor therapy (22.2%), as shown in Figure 3. According to the opinions of PCPs on the best approach to increase knowledge about back pain, 65% believed that a practical session was the best method, 49.5% favored teaching sessions, and 46.6% preferred electronic updates.

**Table 1:** Demographic and work-related characteristics of the physicians.

Physician characteristics	N (%)
Age (yr)	
25–35	35 (34%)
36–45	34 (33%)
46–55	25 (24.3%)
>55	9 (8.7%)
Sex	
Male	54 (52.4%)
Female	49 (47.6%)
Specialty	
General practitioner	49 (47.6%)
Family physician	54 (52.4%)
Working place	
Makkah	21 (20.4%)
Jeddah	31 (30.1%)
Madinah	27 (26.2%)
Yanbu	7 (6.8%)
AIula	3 (2.9%)
Taif	14 (13.6%)
Years of experience	
<5	27 (26.2%)
5–10	22 (21.4%)
>10	54 (52.4%)
Frequency of cases of back pain seen/week	
<5	41 (39.8%)
5–9	46 (44.7%)
≥10	16 (15.5%)

**Table 2:** History questions that patients presenting with inflammatory back pain were frequently asked by physicians.

Variables	N (%)
Psoriasis	56 (54.4%)
Uveitis	64 (62.1%)
Inflammatory bowel disease	82 (79.6%)
Genitourinary/gut infection in the last 1 mo	66 (64.1%)
Enthesitis	44 (42.7%)
Dactylitis	53 (51.5%)
Peripheral arthritis	95 (92.2%)
Family history of spondyloarthritis	86 (83.5%)

**Table 3:** Important investigations in cases of suspicion of inflammatory back pain as reported by primary care physicians.

Investigations	N (%)
CRP/ESR blood test	89 (86.4%)
HLA-B27 blood test	49 (47.6%)
Radiography of the pelvis	49 (47.6%)
Radiography of the spine	68 (66%)
MRI of the spine	48 (46.6%)
MRI of the sacroiliac joint	36 (35%)

CRP = C-reactive protein, ESR = erythrocyte sedimentation rate, HLA = human leukocyte antigen, MRI = magnetic resonance imaging.

## 4. Discussion

To the best of our knowledge, this is the first study to assess the awareness of PCPs regarding the disease characteristics and treatment of IBP in Saudi Arabia. The PCPs reported that improvement with rest, acute onset, morning stiffness, alternating buttock pain, positive family history, improvement with exercise, and duration of > 3 months were significant and more differentiable in patients suspected of having IBP, whereas the age of onset, improvement with NSAIDs, and improvement with rest were not. Numerous studies have shown that questioning one SpA-related condition is not useful for primary care diagnosis, and combinations of several SpA questions are more helpful.^[19]^ For example, NSAIDs, the most frequently prescribed medications for low back pain globally, are used because of their analgesic and anti-inflammatory properties.^[20]^ Unfortunately, no established method for SpA detection has been proven useful in primary care. However, attempts have been undertaken to develop assessment algorithms and a streamlined referral method for primary care patients with spondyloarthropathy.^[8,21]^ Thus, referral strategies are intended to be used for patients who have experienced back pain for three months or more and are less than 45 years at the time of initiation, a group of people referred to as at-risk individuals.^[19,22,23]^ In our study, the PCPs were not fully aware of the related characteristics of SpA. While some may argue that investigating additional SpA characteristics is beyond the scope of the responsibility of a PCP, awareness of the disease spectrum would undoubtedly help identify patients who do not manifest typical IBP symptoms and increase the index of clinical suspicion for considering a diagnosis of SpA. Our data highlighted the need to enhance the awareness of PCPs. According to the PCPs included in our study, whole-spine X-rays and CRP blood tests were considered the most crucial investigations for individuals with suspected IBP. In a survey by Moorthy et al^[24]^ 97% of 151 GPs believed that CRP was an important investigation. However, CRP levels are high in fewer than half of those with spondyloarthropathy.^[24]^ Additionally, individuals with AS and IBP might take up to 10 years to show radiographic abnormalities.^[25]^ Furthermore, according to the current National Institute for Health and Care Excellence guidelines, whole-spine radiography is not recommended for diagnosing low back pain.^[26]^ Therefore, although almost half of the PCPs were aware of NSAIDs and the usefulness of physiotherapy, there was a lack of knowledge regarding the availability and usefulness of biological treatments for IBP. Cooperation and co-management with rheumatologists are crucial parts of axSpA treatment. Thus, knowledge regarding anti-tumor necrosis factor medication and its adverse effects is critical for maintaining and improving the patient’s overall health status.^[27]^ Our study found several discrepancies in the surveyed PCPs’ perceptions of the diagnosis and management of IBP. For example, PCPs were aware of the difference between MBP and IBP but could not exactly ascertain how to distinguish them. In addition, they expressed more confidence in managing MBP. PCPs serve as the gateway to rheumatology for the evaluation and long-term care of axSpA. Therefore, any proposed strategy for PCPs to detect patients with axial SpA should be able to be feasibly implemented in daily practice. Different screening and referral strategies have been proposed to assist PCPs in determining the consideration of an axSpA diagnosis and facilitating the early assessment of suspected axSpA. to assist PCPs in determining the consideration of an axSpA. The Multicenter Ankylosing Spondylitis Survey Trial to Evaluate and Compare Referral Parameters in Early SpA (MASTER) study assesses two referral strategies for at-risk patients: those with at least 3 months of low back pain and less than 45 years old at onset. According to Strategy 1, appropriate individuals are referred if they meet at least one of the following screening criteria: IBP, positive HLA-B27, or imaging-detected sacroiliitis. Patients are referred to Strategy 2 if two out of five criteria are met: the three criteria from Strategy 1 plus a family history of AS or good response to NSAIDs.^[8]^ Similar strategies have been examined in research globally, including extra-articular symptoms, such as uveitis, psoriasis, and IBD, as per Strategy 2.^[23]^ Patients referred by Strategy 1 (35.6%) and Strategy 2 (39.8%) had AxSpA, and IBP was the most often utilized referral parameter across both strategies (93% and 96%, respectively). Similarly, the ASAS recommends a lengthy referral strategy to maximize sensitivity.^[15]^ A previous study assessed 13 referral strategies in a SPondyloArthritis Caught Early cohort.^[28]^ Although enhanced sensitivity is associated with lower specificity, the ASAS approach effectively ensures that no patient with axSpA is missed. Alternatively, if a more stringent method is required, such as restricting the number of referrals, the MASTER technique could be implemented. Because of variances in healthcare systems and referral requirements, the ideal referral strategy differs across countries.

Our study has several limitations. First, it had a relatively small sample size; second, the study method was a survey. Therefore, the PCPs who replied may have been a self-selecting group with a keen interest in chronic back pain, which would have biased and restricted the generalizability of our findings. However, the main goal of this study was to assess the depth of awareness and understanding rather than extend the existing findings to all GPs. Our results imply that IBP requires more attention using future educational programs. In addition, there is a need to establish care pathways for patients with LBP in Saudi Arabia to assist PCPs in determining whether to refer patients to higher-level centers to avoid unnecessary referrals and increased expenses.

## 5. Conclusion

This study showed that PCPs had modest levels of confidence when assessing IBP. Nevertheless, additional research is necessary to evaluate whether these results reflect a need for education or a lack of performance regarding these questions among PCPs. Additionally, simpler evaluation algorithms should be developed and prospectively verified using the criteria known to PCPs. Finally, appropriate SpA training and educational sessions appear to be a primary care education requirement that has not been fulfilled.

## Acknowledgments

We would like to express our gratitude to the Saudi Commission for Health Specialties for their cooperation in distributing the questionnaire.

## Author contributions

**Formal analysis:** Roaa Aljohani.

**Supervision:** Roaa Aljohani.

**Writing – original draft:** Noha Yahya Barradah, Amnah Talal Kashkari.

**Writing – review & editing:** Roaa Aljohani.
